# Persistent, Pruritic, Reddish-Brown Papules and Plaques: An Atypical Presentation of Adult-Onset Still’s Disease

**DOI:** 10.7759/cureus.35587

**Published:** 2023-02-28

**Authors:** Carli Whittington, Rebecca Lapides, Keith Morley, Laura Greene

**Affiliations:** 1 Department of Medicine, Division of Dermatology, University of Vermont Medical Center, Burlington, USA; 2 Pathology and Laboratory Medicine/Dermatopathology, University of Vermont Medical Center, Burlington, USA

**Keywords:** diagnosing still disease, atypical still disease, atypical adult-onset still disease, adult-onset still disease, still disease

## Abstract

Adult-onset Still's disease (AOSD) is a systemic inflammatory condition characterized by recurrent fevers and a dermatologic eruption. The eruption is classically described as migratory and evanescent, composed of salmon-pink to erythematous macules, patches, and papules. However, a much rarer skin rash can also be seen in the setting of AOSD. This eruption has a different morphology, appearing as fixed, extremely pruritic papules and plaques. The histology of this atypical form of AOSD is distinct from that of the more common evanescent eruption. Management of AOSD is multi-faceted, aimed at controlling both the acute and chronic phases. Increased awareness of this more uncommon cutaneous presentation of AOSD is vital so that the appropriate diagnosis can be rendered. Herein, the authors describe an atypical presentation of AOSD in a 44-year-old male patient who presented with persistent, pruritic, brownish papules and plaques on the trunk and extremities.

## Introduction

Adult-onset Still's disease (AOSD) is a systemic inflammatory condition typically characterized by recurrent fevers accompanied by an asymptomatic, salmon-pink to erythematous eruption composed of evanescent macules, patches, and papules. While additional symptoms such as pharyngitis, symmetrical myalgias and arthralgias, and lymphadenopathy are common, not all cases present with these systemic symptoms. Herein, the authors describe an atypical cutaneous presentation of AOSD in a 44-year-old male who presented with fevers, arthralgias, and a persistent and extremely pruritic eruption involving his trunk and extremities. The histology of this atypical form of AOSD is distinctive from that of the more common evanescent eruption. Given that AOSD can have high morbidity due to extremely debilitating arthralgias and myalgias, and may be difficult to diagnose, it is important to appreciate both the typical and atypical cutaneous presentations of this multi-organ systemic disease so that the correct diagnosis can be made promptly and treatment initiated.

## Case presentation

Our patient is a 44-year-old male with a past medical history significant for epilepsy, migraines, and well-differentiated invasive rectal adenocarcinoma developing as a 14mm polyp with extensive high-grade dysplasia status-post polypectomy. The patient was on zonisamide 300mg daily for epilepsy, amlodipine 10mg daily and amitriptyline 10mg daily for preventive headache treatment, rizatriptan 10mg daily and medical marijuana for abortive headache treatment, as well as acetaminophen and ibuprofen for arthritic pain.

**Figure 1:**
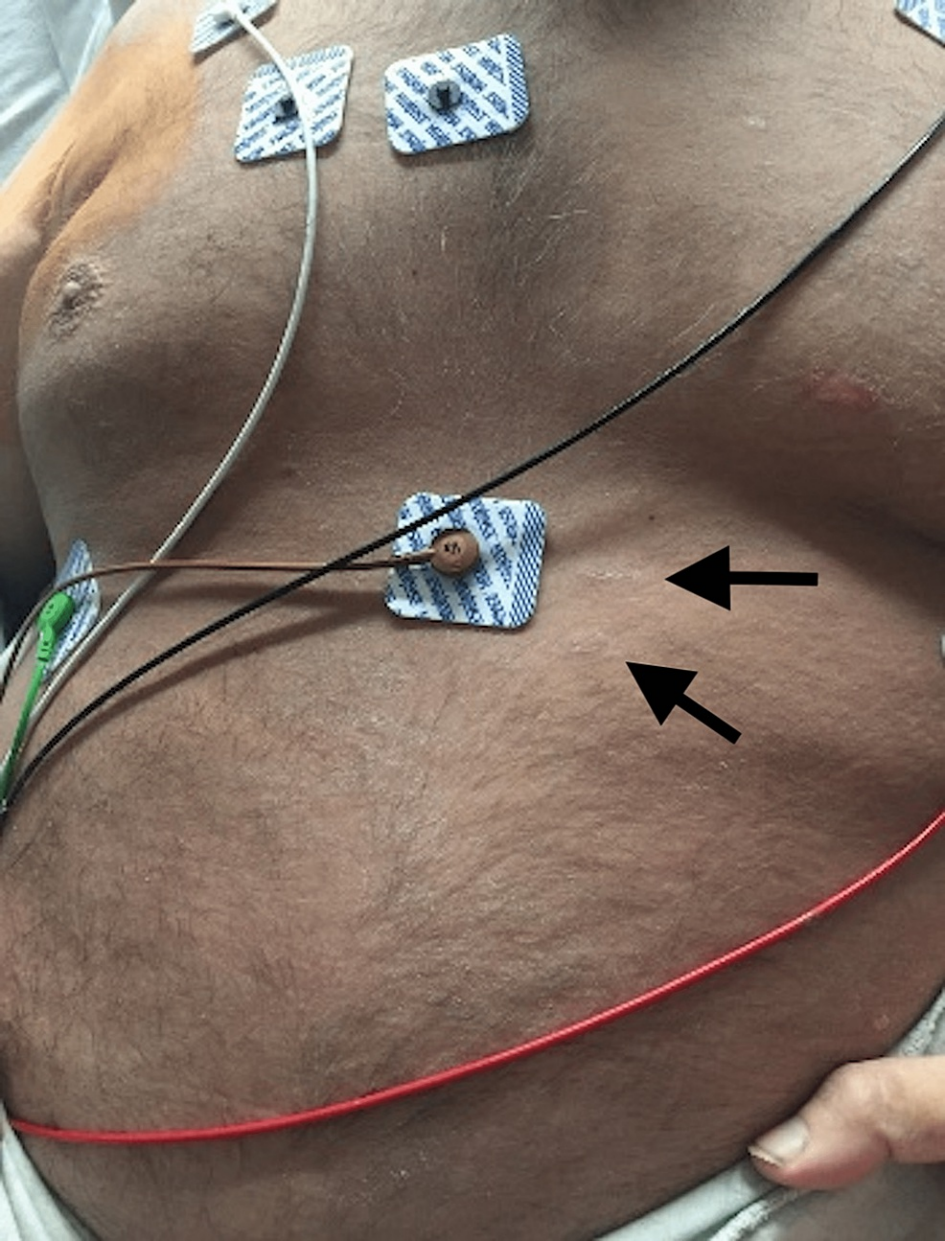
Broad, thin, velvety, and reddish-brown to hyperpigmented papules and plaques with fine overlying scale

The patient presented with an extremely pruritic rash involving the trunk and extremities, which had been present for approximately 12 months. He reported numerous associated symptoms that began at least 3-6 months prior to presentation, including recurrent spiking fevers ≥ 39°C, shortness of breath, myalgias, bilateral polyarthralgias of multiple large and small joints, progressive and incapacitating muscle weakness, and unintentional weight loss of about 22.7 kilograms (50 pounds). Dermatology was consulted. Physical examination revealed broad, thin, velvety, and reddish-brown to hyperpigmented papules and plaques with fine overlying scale (Figure 1) with secondary linear excoriations. There was bilateral axillary lymphadenopathy. There was no ocular, oral, or other mucosal site involvement. A 4-mm punch biopsy was obtained of the left upper back, which revealed findings most consistent with the persistent papules and plaques variant of AOSD, a much more rare rash associated with this condition. The histopathologic findings ruled out other possible differential diagnoses, including connective tissue diseases (systemic lupus erythematosus, dermatomyositis), drug-related eruption, acanthosis nigricans, and superficial dermatophytosis infection.

**Figure 2:**
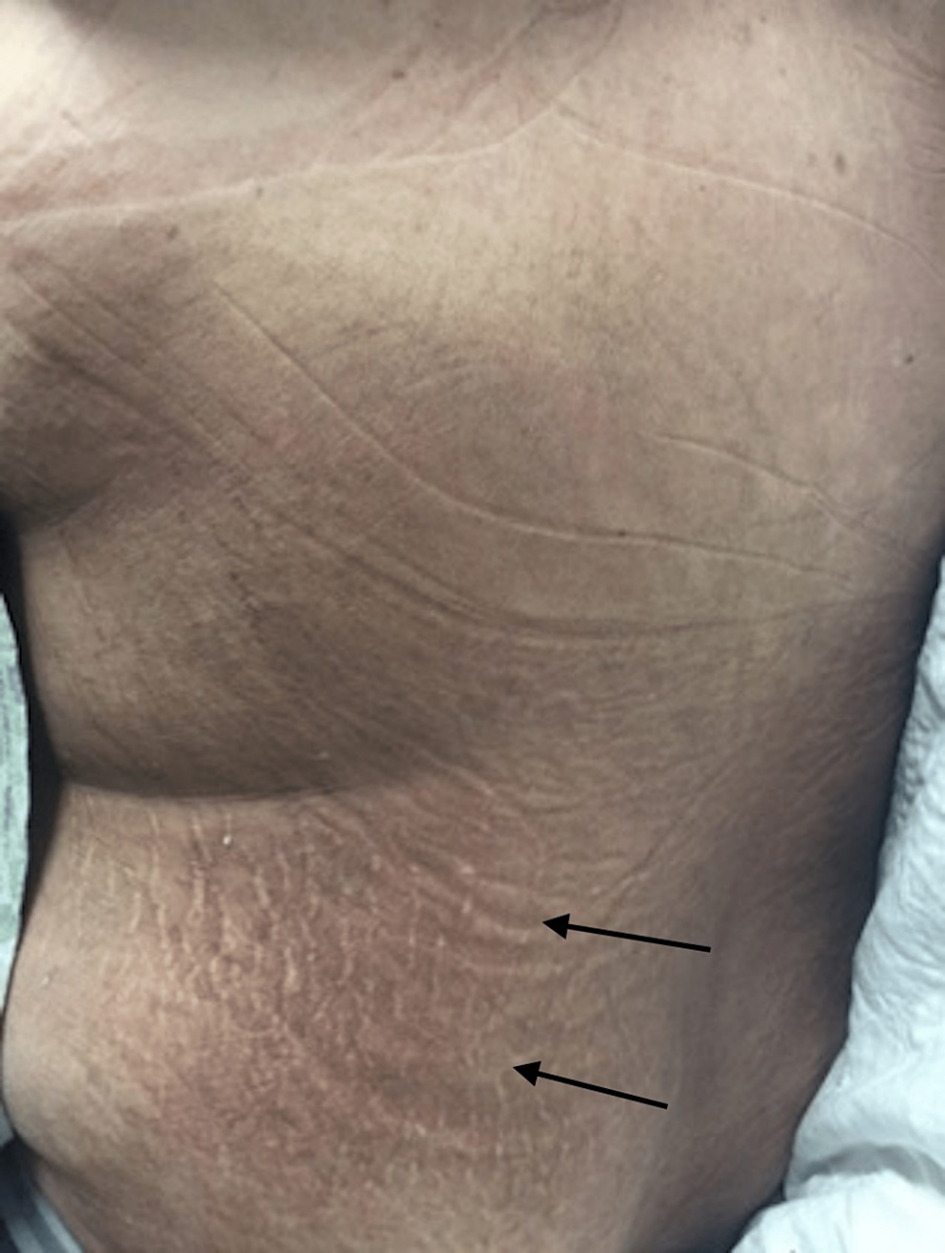
Broad, thin, velvety, and reddish-brown to hyperpigmented papules and plaques with fine overlying scale with secondary linear excoriations.

**Figure 3:**
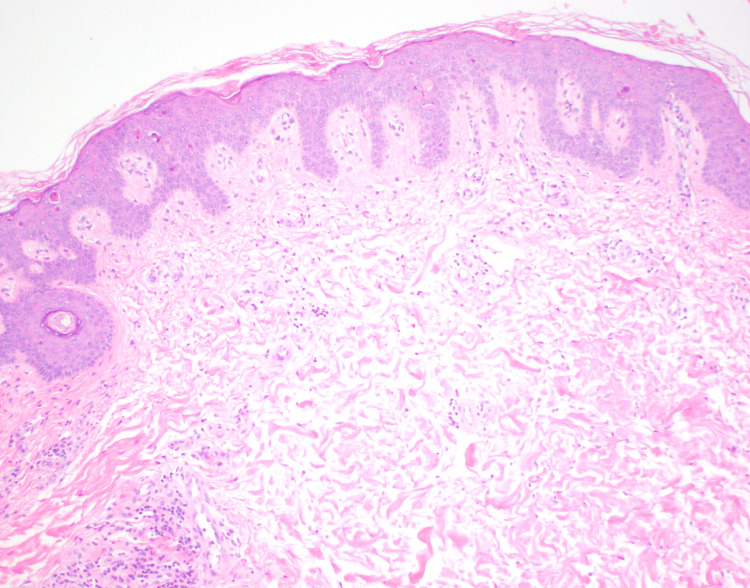
Superficial to mid-dermal perivascular and interstitial mixed inflammatory infiltrate composed of lymphomononuclear cells, neutrophils, and eosinophils. Magnification 10x H&E.

**Figure 4:**
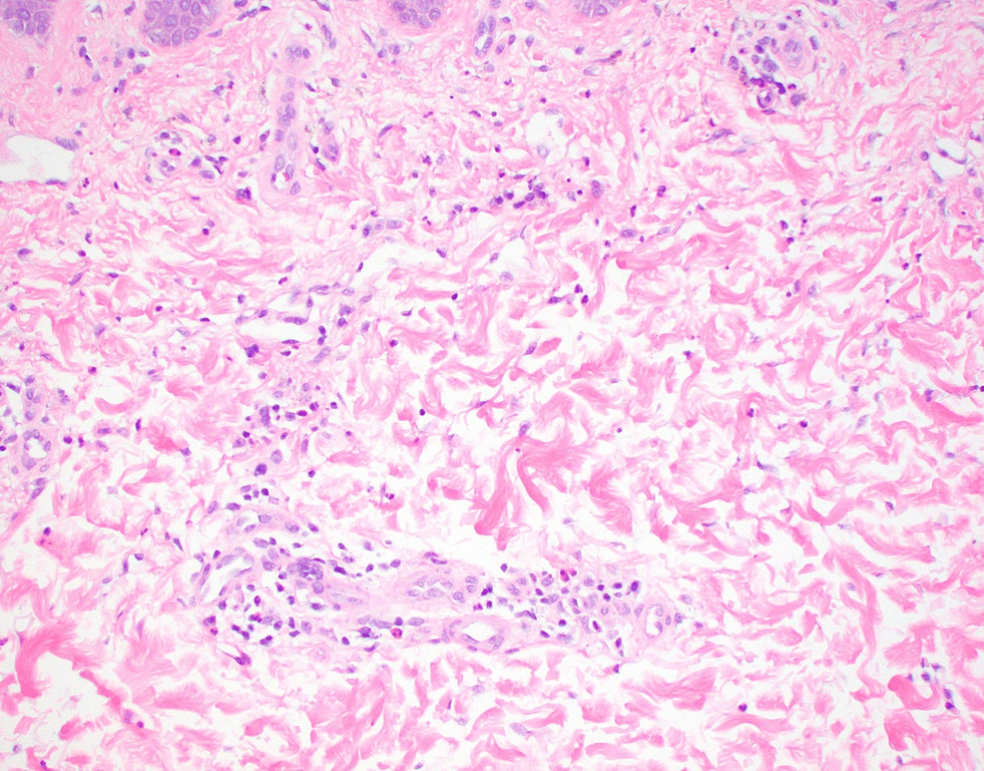
Superficial to mid-dermal perivascular and interstitial mixed inflammatory infiltrate composed of lymphomononuclear cells, neutrophils, and eosinophils. Magnification 20x H&E.

A histopathologic assessment revealed a superficial to mid-dermal perivascular and interstitial mixed inflammatory infiltrate composed of lymphomononuclear cells, neutrophils, and eosinophils (Figure 2-3). There were subtle foci of basal zone vacuolization present. Necrotic keratinocytes were observed at all layers of the epidermis, including within the stratum corneum (Figure 4). Infectious (bacterial, fungal, and atypical mycobacterial) stains were negative. Colloidal iron stain revealed only minimal to mild mucin within the dermis.

**Figure 5:**
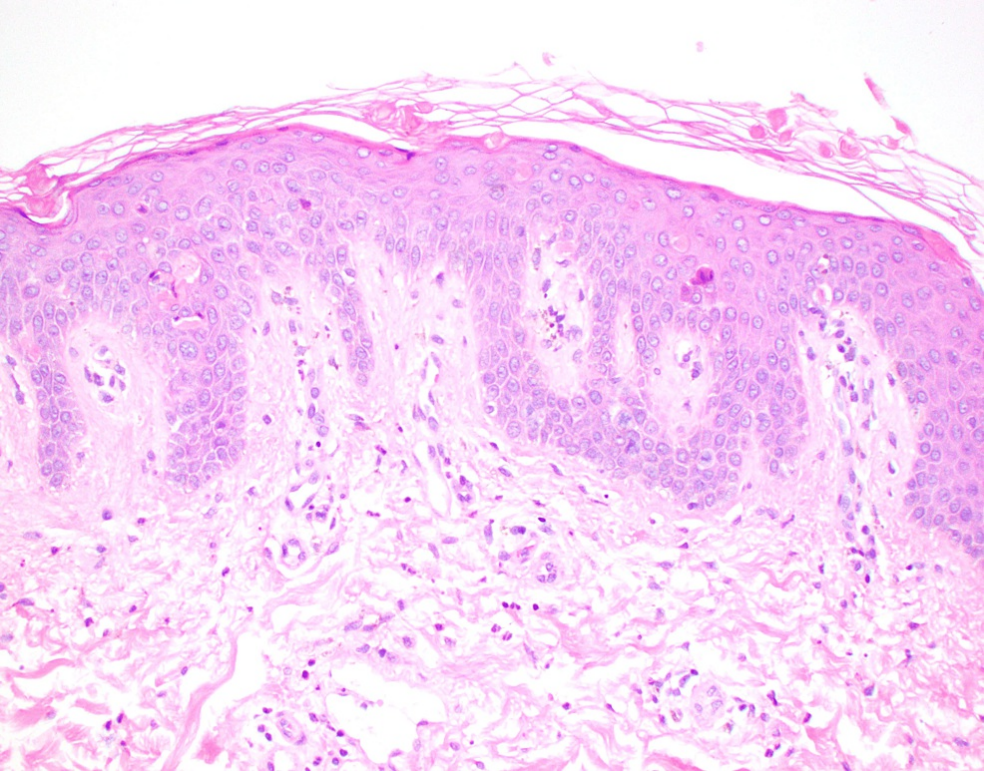
Necrotic keratinocytes in all layers of the epidermis. Magnification 20x H&E.

Initial laboratory work-up showed leukocytosis (white blood cell count > 36,000 with granulocytosis), anemia (Hgb 7.9 gm/dl, reference range 13.3-17.3), thrombocytosis (Plt 577 K/cm^3^, reference range 141-377), elevated erythrocyte sedimentation rate (99 mm/hr, reference range 0-15), elevated C-reactive protein (340.9 mg/L, reference range <10), elevated lactate dehydrogenase (1450 U/L, reference range 313-618), and hyperferritinemia (ferritin >6000 ng/dl, reference range 22-322). Liver function tests were within normal limits and carcinoembryonic antigen, antinuclear antibody, antinuclear cytoplasmic antibody, anti-cyclic citrullinated peptide antibody, and rheumatoid factor tests were all negative. Extensive infectious disease evaluation was negative. Radiologic imaging was positive for axillary and mediastinal lymphadenopathy, as well as edema involving the lumbar and gluteal musculature. No organomegaly was detected. Right axillary lymph node ultrasound-guided fine needle aspiration and core needle biopsy showed findings supportive of a reactive lymph node. Out of concern for myositis, a muscle biopsy was performed but did not reveal an inflammatory myopathy. Flow cytometry was negative for clonal lymphoproliferative or myeloproliferative disorders.

## Discussion

Adult-onset Still's disease (AOSD) is a rare, idiopathic, systemic inflammatory condition that most commonly affects individuals in a bimodal pattern, typically between the ages of 15-25 years and 36-46 years [1]. In some series, there is a female predominance. The typical presentation involves recurrent spiking fevers, arthritis, and an evanescent rash. Macrophage activation syndrome is the more severe complication of AOSD due to the high mortality rate and has been reported in up to 15% of cases. AOSD is often difficult to diagnose due to its non-specific presenting symptoms. AOSD is considered a diagnosis of exclusion, as infectious, malignant, and other autoimmune inflammatory etiologies must be ruled out first [1]. The exact pathogenesis of AOSD has not yet been fully elucidated; however, it is hypothesized that AOSD develops due to a combination of genetic predisposition (especially certain human leukocyte antigens) and a trigger that an individual is exposed to, namely an infectious one. Of note, a specific infectious trigger has not yet been consistently associated with AOSD in the medical literature [1]. Interleukin-1 (IL-1) cytokines are also believed to play a role in the development of AOSD because these are often found to be elevated in patients ultimately diagnosed with AOSD.

Clinicaly, AOSD is characterized by recurrent, high-spiking fevers ≥ 39°C that tend to peak in the afternoon or early evening, then resolve within several hours. Symptoms such as pharyngitis, symmetrical myalgias and arthralgias, and lymphadenopathy are common. Rarely, AOSD can involve internal organs, such as the liver, and even less commonly, the lungs. There can be associated underlying malignancies including lymphomas, solid tumors, and hematopoietic cancers [1-5]. Dermatologically, patients with classic AOSD present with an asymptomatic, salmon-pink to erythematous eruption comprised of evanescent macules, patches, and papules. This eruption tends to appear during fever spikes, but then disappears once the fevers resolve. Most commonly, the rash involves the trunk, but can spread to the upper and lower extremities [2].

Different classification criteria exist to diagnose AOSD, which are a combination of clinical and laboratory findings. Although all have limitations, the criteria described by Yamaguchi are considered the most sensitive. This involves a set of both major and minor criteria, wherein to be diagnosed with AOSD, a patient must be positive for a total of five features including at least two major features. Major criteria in the Yamaguchi system include fever ≥ 39°C lasting ≥ 1 week, arthralgias of ≥ 2 weeks duration, non-pruritic salmon-pink rash of macules and papules involving the trunk (with or without the extremities) that corresponds with febrile episodes, and leukocytosis (≥ 10,000 white blood cells and ≥ 80% granulocytes). Minor criteria in the Yamaguchi system include pharyngitis, lymphadenopathy, hepatomegaly or splenomegaly, elevated liver function tests, elevated lactate dehydrogenase, negative antinuclear antibody, and negative rheumatoid factor [6,7].

The histologic features of the classic eruption of AOSD are a superficial, perivascular dermal inflammatory infiltrate comprised of lymphocytes and neutrophils with minimal to no epidermal changes [8,9]. The persistent papules and plaques variant of AOSD is considered “atypical” because of its distinct physical examination features and histopathologic findings that are dissimilar from classic AOSD [10,11]. Clinically, atypical AOSD presents as violaceous to reddish-brown, scaly, and pruritic papules and plaques on the trunk and extremities. This eruption can have a rippled appearance and tends to persist independent of the febrile episodes [12]. A distinguishing and unique histologic feature of atypical AOSD is the presence of dyskeratotic keratinocytes in the upper one-third of the epidermis, although these necrotic cells can involve all layers of the epidermis. Dyskeratotic cells are also noted in the stratum corneum [7,9]. Basal zone vacuolization, eosinophils, and mucin deposition have been reported [8,13]. Ultimately, to accurately diagnose the atypical variant of AOSD, the clinical, laboratory (serologic), and histopathologic features should all be closely correlated.

Treatment of AOSD usually involves a two-headed approach, wherein the initial step is to control the acute systemic features and the second step is to maintain long-term disease control. Depending upon which organ systems are involved and/or if an underlying malignancy is present, a multi-disciplinary team of specialists may need to be consulted. Acute disease control commonly involves the initiation of high-dose oral non-steroidal anti-inflammatory drugs (NSAIDs) or aspirin with or without oral prednisone. An alternative, second-line agent that can be used is oral methotrexate. To achieve long-term disease control, newer agents are reported to have some success, including anakinra (an IL-1 receptor antagonist), tocilizumab (an IL-6 receptor antagonist), tumor necrosis factor-alpha inhibitors (TNF-alpha blockade), and rituximab (an anti-CD20 antibody) [1].

In regards to the patient reported herein, he met sufficient major and minor Yamaguchi criteria for the diagnosis of AOSD. Positive major criteria include fevers ≥ 39°C lasting ≥ 1 week, arthralgias of ≥ 2 weeks duration, and leukocytosis (≥ 10,000 white blood cells with ≥ 80% granulocytes). Positive minor criteria include lymphadenopathy, elevated lactate dehydrogenase, negative antinuclear antibody, and negative rheumatoid factor. Clinically, he did not present with the classic asymptomatic, salmon-pink, evanescent maculopapular cutaneous eruption. Instead, the patient’s rash consisted of persistent, pruritic, reddish-brown papules and plaques. When a skin biopsy was performed, histopathologic examination revealed findings most consistent with the persistent papules and plaques variant of AOSD rather than classic AOSD (Figure 1). Dermal inflammation (Figure 2) as well as dyskeratotic keratinocytes (Figure 3) that are characteristic of atypical AOSD can be appreciated in the specimen. While other entities such as connective tissue diseases (systemic lupus erythematosus, dermatomyositis), drug-related eruption, acanthosis nigricans, and superficial dermatophytosis infection were included in the initial differential diagnosis, the histopathologic findings were not consistent with any of these conditions when correlated with the clinical features and serologic results. The patient was started on oral prednisone and NSAIDs, and then gradually transitioned to Anakinra, which resulted in good control of his skin pruritus, fevers, myalgias, polyarthragias, and other systemic symptoms. No new underlying malignancy was detected and there has been no recurrence of his adenocarcinoma of the colon to date, which was detected approximately 2-3 years prior to AOSD diagnosis.

## Conclusions

Adult-onset Still's disease is an uncommon systemic inflammatory condition characterized by recurrent fevers and an associated pruritic dermatologic eruption that can be classified as typical or atypical. The atypical clinical presentation of AOSD can pose a diagnostic challenge for the clinician, as it differs drastically from the more common salmon-pink, evanescent patches and plaques that characterize typical AOSD. Given this, there is potential for delayed diagnosis and postponed treatment initiation for these patients, which can be extremely problematic given that underlying malignancy may be present. Furthermore, achieving disease remission and then maintaining adequate maintenance therapy is critical, and the determination of the appropriate regimen may take time. This rare case serves to increase awareness of the less common presentation of persistent pruritic hyperpigmented papules and plaques of atypical AOSD correlated with the unique histopathologic features.
